# A Xenogeneic-Free Protocol for Isolation and Expansion of Human Adipose Stem Cells for Clinical Uses

**DOI:** 10.1371/journal.pone.0067870

**Published:** 2013-07-09

**Authors:** Carmen Escobedo-Lucea, Carmen Bellver, Carolina Gandia, Andres Sanz-Garcia, Francisco J. Esteban, Vicente Mirabet, Giancarlo Forte, Isabel Moreno, Melissa Lezameta, Angel Ayuso-Sacido, José M. Garcia-Verdugo

**Affiliations:** 1 Comparative Neurobiology Unit, Instituto Cavanilles, University of Valencia- RETICS, Valencia, Spain; 2 Division of Biopharmaceutics and Pharmacokinetics, Faculty of Pharmacy, University of Helsinki, Helsinki, Finland; 3 Department of Experimental Biology, Systems Biology Unit, University of Jaén, Jaén, Spain; 4 Cell and Tissue Bank, Regional Transfusion Center, Valencia, Spain; 5 Smart Materials Group, National Institute for Materials Science, Tsukuba, Japan; 6 International Clinical Research Center, St. Annes University Hospital, Brno, Czech Republic; 7 Anatomy and Histology Department, Faculty of Medicine, University of Valencia, Valencia, Spain; 8 Integral Oncology Centre Clara Campal and Molecular Applied Medicine Institute, Hospital de Madrid Foundation, Madrid, Spain; University of Sao Paulo - USP, Brazil

## Abstract

Human adipose stem cells (hASCs) play a crucial role in the fields of regenerative medicine and tissue engineering for different reasons: the abundance of adipose tissue, their easy harvesting, the ability to multipotent differentiation and the fact that they do not trigger allogeneic blood response or secrete cytokines that act as immunosuppressants. The vast majority of protocols use animal origin reagents, with the underlying risk of transmitting infections by non-human pathogens. We have designed a protocol to isolate and maintain the properties of hASCs avoiding xenogeneic reagents. These changes not only preserve hASCs morphology, but also increase cell proliferation and maintain their stem cell marker profile. On the other hand, human serum albumin (HSA), Tryple® and human Serum (HS), do not affect hASCs multipotent differentiation ability. The amendments introduced do not trigger modifications in the transcriptional profile of hASCs, alterations in key biochemical pathways or malignization. Thus, we have proven that it is possible to isolate and maintain hASCs avoiding animal reagents and, at the same time, preserving crucial culture parameters during long term culture. Thereby we have revealed a novel and effective tool for the improvement of clinical, cell-based therapies.

## Introduction

Mesenchymal stromal cells (MSCs) have been the most widely used in preclinical and clinical assays so far[1]–[7]. MSCs can be obtained from a variety of tissues [8]–[10], including the stromal non-hematopoietic fraction of the bone marrow and adipose tissue [11], [12]. MSCs from bone marrow (BM-MSCs), have been thoroughly described and characterized since they were the first adult stem cell type identified and isolated [13]. A large number of studies have analyzed the fate of adult stem cells administered *in vivo* as well as the possible mechanisms by which they might operate in the treatment of different diseases [6], [14]–[18]. In most procedures, isolated stem cells would need to be expanded *in vitro* to obtain the number of cells required for clinical efficiency. However, *in vitro* expansion increases the potential risk of contamination and can also affect cell survival and function. Among the MSCs obtained from other sources, human adipose stem cells (hASCs) have emerged as strong candidates to play a crucial role in the fields of regenerative medicine and tissue engineering for several reasons. They can be easily harvested from fat tissue, which is an abundant source. The cell yield per gram of tissue is 500-fold that obtained for BM-MSCs [19], [20]. They show high rate of proliferation *in vitro* [12], [21] and have the ability to differentiate into several lineages: osteogenic, chondrogenic, adipogenic and myogenic cells [11]. In addition, hASCs, like their bone marrow counterparts, do not trigger the response of allogeneic lymphocytes *in vitro* [22], [23]. hASCs have also proven to be successful, also for their ability to secrete cytokines, act as immunosuppressants and activate antioxidant and angiogenic pathways [20], [24].

A number of clinical trials involving the treatment of a variety of diseases hASC is already ongoing [14], [22], [25]–[29].

One of the key points for their use in clinics regards on the cell isolation and expansion procedures. The vast majority of protocols use reagents derived from animal sources, with the underlying risk of transmitting infections by non-human pathogens [30], [31].

Use of bovine derivatives (serum as supplement for cell growth medium or serum albumin for cell washing saline solution) can result in bacterial, viral and prion infections [32]–[36], or triggering of an immunological reaction mediated by serum bovine proteins [37], [38]. Although little is known about other xenogeneic products, as porcine-derived trypsin used for detaching cultured cells, those products are likely to induce similar biosafety risks. The objective of our research was to design a protocol to isolate, expand and maintain clinically safe and efficient hASCs, avoiding xenogenic reagents. With this aim we replaced all animal reagents used for isolation and maintainance by human or synthetic derivatives. To validate the amendments, we compared the results obtained after introduce the amendments, as well as gene expression profile of the cells isolated and maintained in xenogeneic conditions.

## Methods

### Cell Harvesting and Culture Conditions

Human adipose tissue samples were obtained at private plastic surgery clinic (Clinica Dra. Isabel Moreno) from lipoaspiration procedures from 8 healthy patients under surgery by aesthetic or beauty reasons (two of them were man and the rest were woman), aged between 18 and 35, following written informed consent and ethical research project approval by both Clinica Dra Isabel Moreno and Principe Felipe Research Center (CIPF) ethical boards. All the patients were previously screened for human immunodeficiency virus (HIV), hepatitis C and other infectious diseases. Cells were obtained following the protocol established from Planat-Benard [7], with a few modifications. Briefly, samples were digested in a solution of 1 mg/ml collagenase type I from Clostridium Histolyticum (Gibco, Grand Island, NY) for 90 minutes at 37°C. The cells were then washed with 0.5% of HSA in Hank’s balanced salt solution (Gibco, Grand Island, NY) and after discarding mature adipocytes, seeded in culture flasks with growth medium, Dulbecco’s modified Eagle’s medium (Invitrogen) supplemented with human or bovine serum mesenchymal stem cell qualified (Gibco, Grand Island, NY), in a humidified atmosphere of 95% air and 5% CO_2_ at 37°C. The medium was replaced every 3 days. When primary culture became subconfluent, cells were detached using Tryple® (Invitrogen) and subcultured in growth medium.

### Serum Collection and Storage

Human serum (HS) group A was obtained from voluntary healthy blood donors at the Valencia Regional Transfusion Centre. Donors were screened for disease risk factors using a health history questionnaire and laboratory tests, fulfilling law requirements for blood donations. Human sera (non lipemic and negative for irregular antibody screening test) were pooled (80–100 donors), centrifuged (2900 g for 10 min) and stored at −30°C, until use.

### Experimental Design and Samples

We established four points of analysis per patient: isolation point, that is, immediately after the isolation procedure; passage 1 (P1), or human adipose adherent cells after their first passage (3–5 days in culture); passage 3 (P3), after 10–12 days in culture); and passage 5 (P5), following the fifth expansion around 20 days after the isolation. This organization scheme was used throughout the entire project for all experiments performed. The total number of patients used in this study was n = 8, from which three were used for the growth curve, proliferation assays and differentiation experiments. Samples from the 8 patients were used for the rest of studies.

### Proliferation Kinetics

To assess the proliferative capacity of hASCs, parallel cultures from three donors were analyzed through eleven serial passages using two different culture conditions: 6% HS and 10% fetal bovine serum (FBS). Cells were counted and subcultured every four days. At each passage, the population doubling (PD) rate was determined using the formula *x = [log_10_(N_H_)- log_10_(N_1_)]/log_10_(2)*, where *N_1_* is the plated cell number and N_H_ is the cell number at harvest [8]. Cumulative population doubling rate was calculated, adding to each passage the PD rate of the previous passages.

A growth curve was carried out in parallel using hASCs from n = 3 donors, starting at passage 3. Two hundred cells per square centimeter were plated in P24 plates (Beckton Dickinson). Every day the cells from two wells were harvested and counted.

### RT-PCR

hASCs from the 8 patients were analyzed for a list of genes summarized on additional Table S1, using RT-PCR techniques. H9 cells (Wicell) and commercial adipose tissue RNA (Stratagene) were used as positive controls. Total RNA were extracted using the RNeasy kit (Qiagen), according to manufacturer’s instructions, and treated with DNAse (Qiagen). Total RNA obtained was checked by spectroscopy using Nanodrop in order to assess the quantity and purity acquired. An *A_260_/A_280_* ratio between 1.8–2.0 was deemed optimal to accept the sample for experimental procedures. Total RNA was then converted to cDNA through reverse transcription using the High Capacity cDNA Reverse Transcription Kit (Applied Biosystems), in which the reaction mixture contains 2 µg of total RNA, 2 µL of RT Buffer 10X, 2 µL of Random Primers 10X, 0.8 µL of dNTPs and 1 µL of enzyme. The reaction was adjusted to reach a final volume of 20 µL using DEPC H2O. PCR using the synthesized cDNA was performed to determine the presence or absence of the different transcripts. PCR was carried out using an Eppendorf PCR machine and B-2 microglobulin, b-actin, or GAPDH were used as internal controls.

### Electron Microscopy Studies

For fine ultrastructural analysis, cells were cultured in chamber slides and then serially washed in a 0.1 M phosphate buffer (PB; pH 7.4) solution, prior to their fixation for Transmission Electron Microscopy (TEM). Fixation was performed in 3% glutaraldehyde solution in PB for 30 minutes at 37°C and postfixed in 2% OsO_4_ in PB. Dehydration was achieved by a graded series of ethanol solutions and a final rinse with propylene oxide (Lab Baker, Deventry, Holland). Finally, plates were embedded in araldite (Durkupan, Fluka) overnight. Following polymerization, embedded samples were detached from the chamber slide and glued to Araldite blocks. Serial semi-thin (1.5 µm) sections were cut with an Ultracut UC-6 (Leica, Heidelberg, Germany), mounted onto slides and finally stained with 1% toluidine blue. Ultrathin (0.07 µm) sections were prepared with the Ultracut and stained with lead citrate. Photomicrographs were obtained under a transmission electron microscope (FEI Tecnai Spirit G2), using a digital camera (Morada, Soft Imaging System,Olympus).

### In Vitro Differentiation Assays

Differentiation assays into adipogenic and chondrogenic lineages were performed for hASCs from all culture conditions at passage 1, from a total of n = 3 donors. The assay was then repeated at passage 7 to determine whether cells differentiation capacities were retained throughout long-term culture, following protocols previously established [11]. Cartilage differentiation was confirmed by histochemical staining using Alcian Blue and RT-PCR screening for cartilage specific genes. Adipogenic differentiation was induced in 100% confluent hASCs cultures with induction media. The developing lipid vacuoles were stained with Oil Red and specific genes for adipogenesis were screened for with RT-PCR.

### Microarray Data Analysis

#### Microarray analysis

Hybridization, washing, staining, and scanning of the arrays was performed according to the manufacturer’s instructions (Agilent Technologies “one –color microarray-based gene expression Analysis, 5.5 Version). The array contained 41000 probes.

#### Array design

We used total RNA obtained from lipoaspirates of eight separate donors (aged 18–35 years). The samples obtained from cell cultures consisted in total RNA isolated from 1.6×10^6^ cells. RNA was obtained using the RNeasy kit (Qiagen), according to manufacturer’s instructions, and treated with DNAse. RNA concentration was measured by spectroscopy using Nanodrop with an A_260_/A_280_ ratio of 1.8. Prior to performing the hybridization procedure Agilent p/n 5188–5977 “one-color microarray-based gene expression analysis”, we did a quality and quantity control assay of total RNA, using electrophoresis in an Agilent Bioanalizer. The RNA Integrity Number (RIN) was between 8.0 and 9.9 in all the samples used for hybridization. Total RNA was standardized among patients by pooling together the different samples for each of the established stages. We established four time points for the analysis (Isolation and passages 1, 3 and 5), using four primary culture RNA preparations from 4 different patients per time point. We repeated the hybridization twice per group.Microarrays from hASC xenogeneic free cells are deposited under GEO number GSE46314.

### Microarray Gene Expression Data Analysis

Statistical analysis was carried out using the R-project software [39] and the appropriate Bioconductor packages [40]. First background correction was performed using the subtract function implemented in the R package named Limma [41]. Then, and in order to remove all the possible sources of variation of a non-biological origin, densitometry values were transformed into a logarithmic binary scale and then, they were normalized between arrays using the normalize *Quantiles* function also implemented in the Limma package.

Statistically significant differences between groups were identified using the empirical Bayes method, also implemented as *eBayes* function in Limma package to moderate the standard errors of the estimated log-fold changes. This results in more stable inference and improved power, especially for experiments with small numbers of arrays. This approach includes the Benjamini-Hochberg (BH) multiple hypotheses test using the Bioconductor multtest package for raw p-value correction to ascertain the false positive rate. Those genes showing a BH p-value<0.05 were selected as de-regulated genes. Functional annotations were carried out using the Database for Annotation, Visualization and Integrated Discovery (DAVID) [42], which allows the searching for blocks of functionally-related genes by different criteria such as the Gene Ontology terms and Kyoto Encyclopedia of Genes and Genomes (KEGG) pathways, among others. Finally, functional Networks were obtained using the Ingenuity Pathway Analysis (IPA; Ingenuity Systems, RedwoodCity, CA) [43], in which the gene symbols and the up- and down-regulated significant genes (Tables 1, 2, 3 and 4) were imported.

**Table 1:** Up-regulated genes between passages involved in significant KEEG pathways.

	Number ofgenes BH<0.05	KEGG Pathway	Genes in KEGG pathway	Fisher exactp-value
Passage 1 versus Isolation	63	ECM-receptor interaction	COL1A2, FN1,SDC1	2.5·10^−3^
		Biosynthesis of unsaturatedfatty acids	ELOVL6, FADS1	2.3·10^−3^
Passage 3 versus passage 1	111	Piruvate metabolism	ACAT2, ALDH1B1, PDHB	1.3·10^−3^
		Butanoate metabolism	E2F5,SMAD2,ANAPC1,CDC16,CDNK2A, ORC4L,TFDP2	1.0·10^−4^
		Cell cycle	ACAT2, ALDH1B1, GLO1, PDHB	1.1·10^−4^
Passage 5 versus passage 3	209	Cell division pathway	BBC3, CHEK1, FAS, THBS1	7.2·10^−3^
		Ribosome	MRPL13, RPL22L1, RPS10, RPLP0	1.7·10^−2^
		Pyruvate metabolism	ACAT2, ALDH7A1, ME1	1.0·10^−2^
		Regulation of actin cytoskeleton	WASF1, CFL2, FGF2, PFN2, RRAS2	3.5·10^−2^

**Table 2:** Down-regulated genes between passage 1 versus isolation which are involved in significant KEEG pathways.

Number of genes BH<0.05	KEGG Pathway	Genes in KEGG pathway	Fisher exact p-value
97	Jak-STAT Signaling pathway	EZR, JAM PIK3R3	1.0·10^−2^
	Leucocyte transendothelial migration	ARGDIB,NTRK2 PIK3R3	1.2·10^−2^
	Neurotrophin signalling pathway	IFNGR1,LIFR, PIK3R3 SPRY1	2.7·10^−3^

**Table 3:** Down-regulated genes between passage 3 versus passage 1 which are involved in significant KEEG pathways.

Number of genes BH<0.05	KEGG Pathway	Genes in KEGG pathway	Fisher exact p-value
136	Neurotrophin signaling pathway	PLCG2, NTRK2, NFKB1, PIK3R3, ARHGDIB	2.1·10^−3^
	Epithelial cell signaling in Helicobacterpylori infection	PLCG2, HBEGF, NFKB1, JAM2	1.5·10^−3^
	Viral myocarditis	HLA-DRB1, FYN, HLA-DMA, HLA-DQA1	1.8·10^−3^
	Cell adhesion molecules (CAMs)	HLA-DRB1, CD34, JAM2, HLA-DMA, HLA-DQA1	2.8·10^−3^
	Asthma	HLA-DRB1, HLA-DMA, HLA-DQA1	1.2·10^−3^
	Antigen processing and presentation	HLA-DRB1, HLA-DMA, HLA-DQA1, B2M	3.2·10^−3^
	Hematopoietic cell lineage	IL1R2, HLA-DRB1, CD34, CD14	3.6·10^−3^
	ErbB signaling pathway	PLCG2, HBEGF, PIK3R3, NRG2	3.8·10^−3^
	Allograft rejection	HLA-DRB1, HLA-DMA, HLA-DQA1	2.3·10^−3^
	Graft-versus-host disease	HLA-DRB1, HLA-DMA, HLA-DQA1ç	2.9·10^−3^
	Type I diabetes mellitus	HLA-DRB1, HLA-DMA, HLA-DQA1	3.6·10^−3^
	Intestinal immune network for IgA production	HLA-DRB1, HLA-DMA, HLA-DQA1	5.6·10^−3^
	Autoimmune thyroid disease	HLA-DRB1, HLA-DMA, HLA-DQA1	6.3·10^−3^
	Leukocyte transendothelial migration	EZR, PLCG2, PIK3R3, JAM2	1.1·10^−2^
	Pathogenic Escherichia coli infection	EZR, FYN, CD14	8.5·10^−3^
	Adipocytokine signaling pathway	NFKB1, POMC, CAMKK2	1.3·10^−2^
	Complement and coagulation Cascades	VWF, F13A1, CFH	1.4·10^−2^

**Table 4:** Down-regulated genes between passage 5 versus passage 3 which are involved in significant KEEG pathways.

Number of genes BH<0.05	KEGG Pathway	Genes in KEGG pathway	Fisher exact p-value
237	Cell adhesion molecules (CAMs)	ITGA9, PTPRM, HLA-DRB1, CD34, CLDN5, HLA-DRB5, HLA-DPB1, JAM2, HLA-DMA, HLA-DQA1, HLA-F	5.5·10^−7^
	Allograft rejection	HLA-DRB1, HLA-DRB5, GZMB, HLA-DPB1, HLA-DMA, HLA-DQA1, HLA-F	2.2·10^−7^
	Graft-versus-host disease	HLA-DRB1, HLA-DRB5, GZMB, HLA-DPB1, HLA-DMA, HLA-DQA1, HLA-F	4.0·10^−7^
	Type I diabetes mellitus	HLA-DRB1, HLA-DRB5, GZMB, HLA-DPB1, HLA-DMA, HLA-DQA1, HLA-F	6.8·10^−7^
	Asthma	HLA-DRB1, IL13, HLA-DRB5, HLA-DPB1, HLA-DMA, HLA-DQA1	1.2·10^−6^
	Autoimmune thyroid disease	HLA-DRB1, HLA-DRB5, GZMB, HLA-DPB1, HLA-DMA, HLA-DQA1, HLA-F	2.7·10^−6^
	Antigen processing and presentation	HLA-DRB1, HLA-DRB5, HLA-DPB1, HLA-DMA, HLA-DQA1, B2M, HLA-F	7.0·10^−5^
	Hematopoietic cell lineage	IL1R2, HLA-DRB1, CD34, CD33, HLA-DRB5, EPOR, CD14	8.8·10^−5^
	Viral myocarditis	HLA-DRB1, HLA-DRB5, HLA-DPB1, HLA-DMA, HLA-DQA1, HLA-F	2.4·10^−4^
	Intestinal immune network for IgA production	HLA-DRB1, HLA-DRB5, HLA-DPB1, HLA-DMA, HLA-DQA1	3.4·10^−4^
	Systemic lupus erythematosus	HLA-DRB1, HLA-DRB5, HLA-DPB1, HLA-DMA, HLA-DQA1	7.8·10^−3^
	Jak-STAT signaling pathway	SPRY1, IL10RA, STAT5A, IL13, EPOR, PIK3R3	1.3·10^−2^
	Aldosterone-regulated sodium reabsorption	HSD11B2, IGF1, PIK3R3	1.4·10^−2^
	ErbB signaling pathway	STAT5A, HBEGF, PIK3R3, NRG2	2.3·10^−2^

## Results

### Effect of Human Serum and Non-animal Reagents in hASCs Isolation and Maintenance

**Figure 1:**
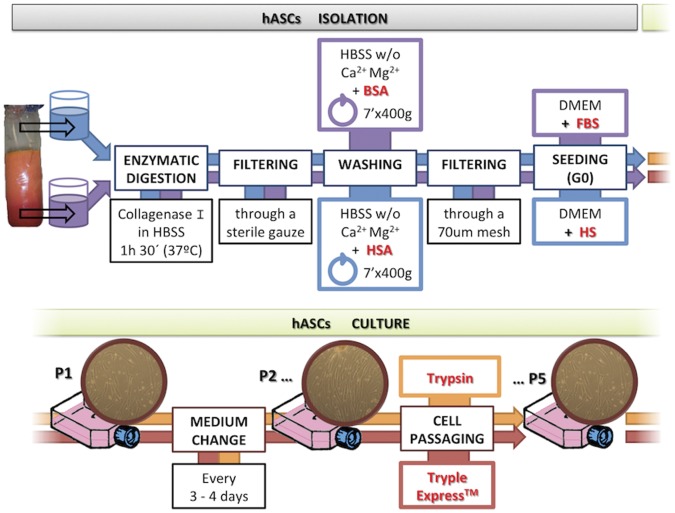
General scheme of the procedure highlighting the modifications introduced.

**Figure 2:**
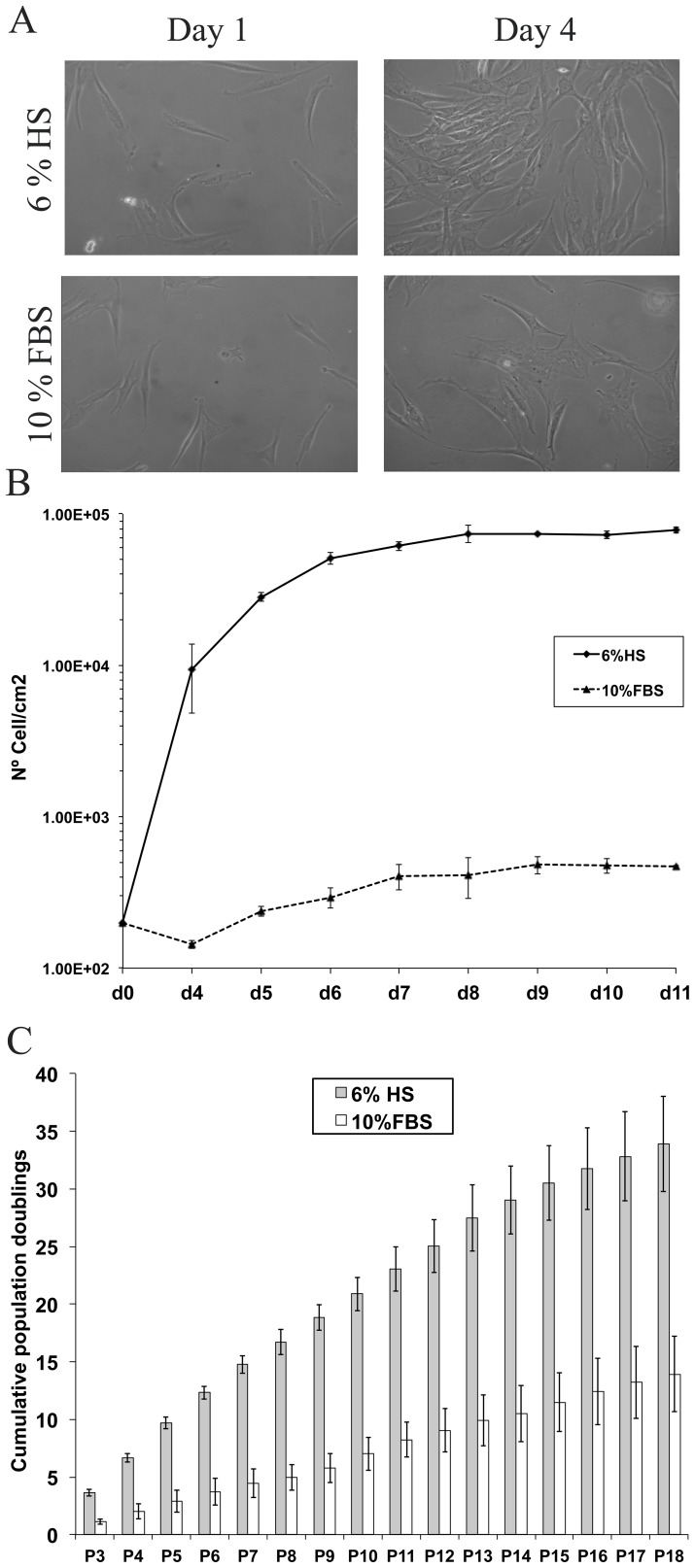
Morphology and expansion capacity of hASCs cultured in 6%HS or 10% FBS. (**A**) Images of hASCs cultures at days 1 and 4 after seeding (200 cells/cm2). Cells were obtained from the same donor and cultured with 6% HS or 10% FBS, respectively (n = 3 donors). All the images were captured at 10X magnification in an Olympus microscope. (**B**) Proliferation kinetics growth curve of hASCs in HS and FBS from seeding until day 11. (**C**) Mean cumulative population doublings for HS (black) and FBS (white). Cells used for both conditions were obtained from the same 3 donors (n = 3) and cultured until passage 18.

Immediately after their obtention in the surgery room, lipoaspirates (≅3 l per patient), were transported to the laboratory and splitted in 2 sterile containers inside a culture hood. For digestion, one was treated with human derivatives (HSA for washing and HS during seeding) and the other with bovine reagents (BSA and FBS). After discard blood derivatives, mature adipocytes and chirurgical infiltration solution, the isolated hASC fractions were seeded under their respective conditions (for summary of the protocol see Fig. 1) to evaluate differences between both protocols. After four days, the cells maintained their characteristic fibroblastic morphology [20], [21]. In contrast, FBS-hASCs showed smaller cellular density, or larger cell size (Fig. 2 A). In order to determine the optimal HS concentration required in the media, alamar blue assay was performed to check cell viability and proliferation. Human serum concentration percentages used were 0, 3, 6 and 10. Based on the growth results obtained, 6% HS concentration was chosen (data not shown).We were using Tryple® for detaching and reseeding the cells.

### Growth Curve and Culture Evolution across Time

Based on previous studies [21], we established a growth curve by seeding passage 3 cells in two groups, with HS or FBS respectively, in a density of 200 cells per square centimeter. Cell counting was performed on a daily basis from day 4 until day 11. Use of HS increased proliferation, which added difficulty for evaluating confluence. HS-hASCs proliferated sooner and faster than those cultured in FBS (Fig. 2 B)(day 0–1 versus day 4 respectively). Concerning the growth curve, the rate of proliferation reached its peak between day 4 (9.3×10^3^±3.7×10^3^ cells/cm^2^ or 3.03 fold the previous measurement) and day 6 (5.1×10^4^±4.1×10^3^ cells/cm^2^ or 1.8 fold) in HS maintained cells. During this period, the population-doubling time (PDT) for cells maintained with HS was less than 24 hours. HS-hASCs began to reach confluence after this point and the rate of proliferation decreased, limited by a cell-density phenomenon, reaching a total of 7.8×10^4^±3.8×10^3^ cells/cm^2^ at day 11. Cells on day 11 were grouped in parallel, an indication that they were entering the plateau phase. In contrast, FBS-hASCs proliferated around day 4 (1.43×10^2^±3.1×10^0^ cells/cm^2^ or 1.65 fold), with a progressive increase in their rate of division until day 7 (4.06×10^2^±7.9×10^1^ cells/cm^2^ or 1.38 fold). The rate of division for FBS-hASCs is lower than that for HS-hASCs. PDT for FBS-hASCs is also longer than for HS-hASCs in the middle of the log phase growth. These results confirm that HS has a high proliferation induction effect.

Concerning proliferation kinetics, comparison of the expansion rates for HS-hASCs and FBS-hASCs showed significant differences (Fig. 2 C). Since the first moment, HS-cultured cells start to show a strong proliferative capacity, with levels of 33.9±4.1 cumulative population doublings at passage 18. At the same time point, FBS-maintained cells reached levels of 13.9±3.3 cumulative population doublings and the proliferation rate decreased immediately. These differences in proliferation kinetics between hASCs cultured on FBS or HS were clear and consistent since the first passage and in all the samples.

### Morphological and Molecular Biology Characterization of hASC Obtained with the Improvements of the Protocol

hASCs were analyzed by PCR for transcriptional evidence of genes associated with adipose mesenchymal stem cells, early development, hematopoietic and adipose tissue markers [see Table S1].

**Figure 3:**
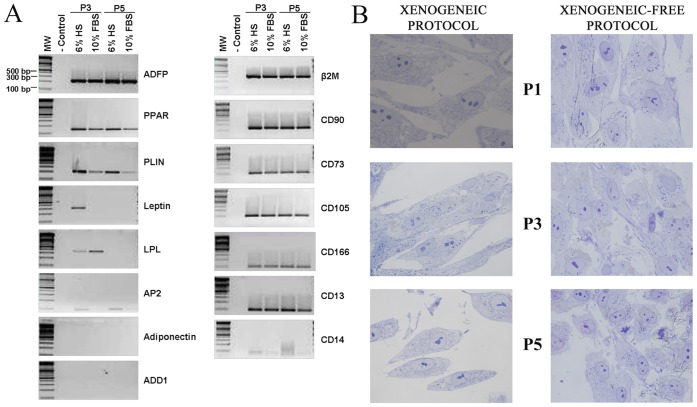
Characterization and comparison of hASC HS/FBS maintained. (**A**) RT-PCR analysis of gene expression in hASCs cultured for 3 and 5 passages on either HS or FBS. Adipose tissue markers or human adipose stem cell markers (respectively). (**B**) Analysis of the morphology of the hASCs cultured under our protocol at passages 1, 3 and 5 respectively. For these structural studies semi-thin sections stained with toluidine blue were used. All the images were captured in a Zeiss Axiovert 200 M microscope. Magnification = 100 X.

We wanted to confirm that cells were expressing markers characteristic of their germ layer and undifferentiated state. We followed the evolution of these markers throughout the culture, analyzing the cells at passages 3 and 5. We first analyzed (Fig. 3 A) the expression of adipose tissue markers. We discarded the mature adipocyte fraction during hASCs isolation process, to make sure that only the stromal vascular fraction was obtained. We searched for specific markers of terminally differentiated adipose cells - Adiponectin, Leptin and Adducin 1 (ADD1) - or genes enrolled in terminal differentiation processes such as fatty acid synthesis and storage of lipid droplets (AP2 and Perilipin, respectively). The results obtained (Fig. 3 A) indicate that with the exception of ADD1, which did not seem to be expressed in any stage of the culture, mature adipose cell markers such as Adiponectin, Leptin, Perilipin and AP2 were clearly expressed following isolation. However, expression levels for these markers gradually decreased throughout maintenance of the culture. Markers for early white adipocyte development and adipocyte progenitor presence such as adipose differentiation-related protein (ADFP), lipoprotein lipase (LPL) and peroxysome proliferator activated receptor (PPAR) were also analyzed. These genes were expressed during the isolation, maintenance and expansion of the cultures (Fig. 3 A). We next analyzed the expression of mesenchymal stem cell markers (Fig. 3 A). Both cells cultured with HS and FBS, were positive for CD90, CD73, CD29, CD105, CD13, CD166. In primary cultures from one sample, cells showing positive results for CD14 and CD45 haematopoietic markers were observed (due to themonocyte/macrophage from peripheral blood). In addition, this culture evidenced the presence of CD34 positive cells from the endothelial lineage. The level of expression of these markers was progressively down regulated (CD14 and CD45) or completely disappearing (CD34) by washings and passages or overgrown by the mesenchymal stromal fraction [See Figure S2]. Flow cytometry was used in parallel to assess the mesenchymal profile. In the absence of animal derivatives, more than 90% of the hASC obtained [see flow cytometry analysis and table in Figure S1] conserve their characteristic surface markers levels for CD90, CD73 and CD44. We did not observe differences between cells cultured with HS or FBS in terms of the percentage of mesenchymal marker expression.

To fully characterize the procedure, a morphological analysis of the cells was also required. Optical microscopy did not reveal major differences between cells cultured with HS versus those cultured with FBS. A more detailed study of the morphology at a semithin section level (Fig. 3 B) allowed us to visualize the evolution of the cells between passages 1, 3 and 5. The majority of the cells in passage 1 exhibit an elongated cellular body, with an oval-shaped nucleus with packed chromatin and several well-developed nucleoli. Their cytoplasm was lightly granular, containing a large number of organelles. At passage 3, cellular morphology was mostly fusiform, but we did observe a reduced number of star-shaped and small cells. The spherical nucleus contained several well-developed nucleoli and lightly-packed chromatin. The cytoplasm was more granular than in passage 1. At passage 5, cellular bodies became polymorphic with a lightly oval-shaped nucleus, characterized by lax chromatin. Mitosis was frequently observed in all 3 stages. We did not observe multinucleated, giant or other abnormal cells.

### Differentiation Ability

**Figure 4:**
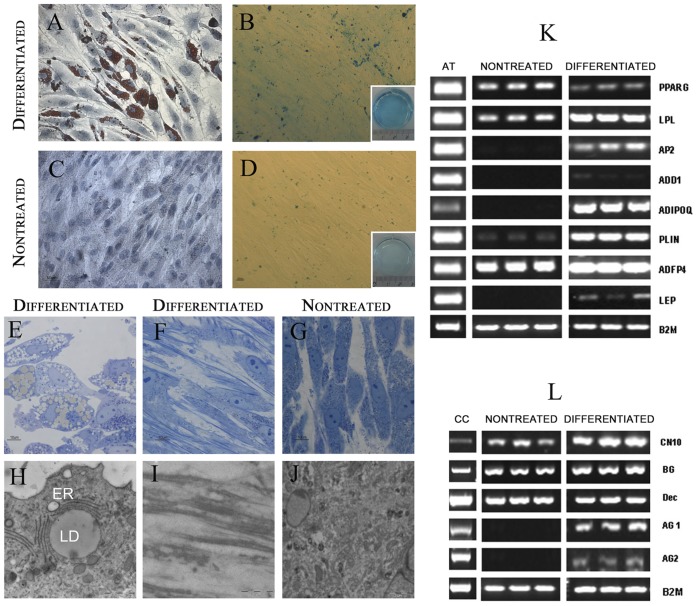
Differentiation ability of HS maintained hASC. Phase contrast images of hASCs subjected to a chemically-defined method for differentiation to adipose and cartilage tissues.Results corresponds to differentiation protocols triggered in cells at 7 passage. (**A**) Oil red O staining of hASCs adipose differentiated cultures on day 12 of differentiation protocol and their corresponding controls, (**B**) Scale bars: 50 µm, (**C**) Alcian-Blue staining for cartilage precursors obtained after 15 days culturing hASCs with specific differentiation protocols or in control media, (**D**) Scale bars: 100 µm. (**E–J**) Transmission electron microscopy characterization of adipocytes (**E, H**), cartilage (**F,I**) and control cells (**G, J**) obtained from hASCs after the differentiation protocol. Scale bars: 5 µm. (**K**) Analysis of adipocyte differentiation markers using RT-PCR. At the time of terminal differentiation culture time, mRNA was prepared for analysis by PCR. Products were visualized by gel electrophoresis. (**L**) Analysis of cartilage differentiation markers using RT-PCR.

To determine whether the lack of animal reagents could diminish this potential, we performed differentiation assays using protocols previously established, to trigger differentiation towards the adipose and chondrogenic lineages [11]. hASCs at passage 3 and 7 were transferred to adipocyte induction media for 10 days and analyzed for lipid accumulation, morphological ultrastructure characterization and RT-PCR for adipose tissue markers. We observed the presence of abundant oil red O-stained lipid droplets in the cytoplasm of cells maintained with adipogenic differentiation media (Fig. 4 A), in contrast to their corresponding controls (Fig. 4 C). Detailed ultrastructure analysis using TEM revealed that their morphology was compatible with mature adipocytes. Semithin sections (Fig. 4 E) allowed us to observe cells with their cytoplasm saturated with lipid droplets and an eccentric nucleus, in contrast to their corresponding controls, fusiform cells with a granular cytoplasm and an oval nucleus (Fig. 4 G, J). Differentiated cells were round and smaller with big lipid droplets, surrounded by a fully developed rough endoplasmic reticle rER (Fig. 4 H). Dense bodies were observed in the cytoplasm of control cells. See (Fig. 4 J). In addition and supporting these observations, differentiated cells expressed the typical gene expression pattern of mature adipocytes (Fig. 4K).

Chondrocyte differentiation was induced with specific media [9] during 15 days. Alcian blue staining confirmed the presence of proteoglycans secreted by cartilage differentiated cells in cultures treated with the differentiation media (Fig. 4 B), but not in their corresponding controls (Fig. 4 D). Semithin sections (Fig. 4 F) showed that differentiated cells were elongated with an eccentric nucleus, generally containing two nucleoli (Fig. 4 F). Fibers were abundant in differentiated cultures, and occasionally formed aggregates (Fig. 4 F). In contrast, controls (Fig. 4 G) conserved their characteristic fusiform shape and lacked the presence of fibers between cells (or around). Electron microscopy studies allowed to observe in detail that the above mentioned fibers in differentiated cells were secreted as extracellular matrix and had a typical collagen organization (Fig. 4 I). Fibers were not detected in control cultures (Fig. 4 J). These results were further confirmed by analysis of gene expression (Fig. 4 L). Differentiated hASCs expressed specific cartilage markers as Aggrecan1 and 2. Expression of other markers such as CN10 or Biglican was increased in differentiated hASCs versus control. No differences in terms of percentage or differentiation ability were observed between hASCs treated on passage 3 versus passage 7.

### Microarray Analysis

**Figure 5:**
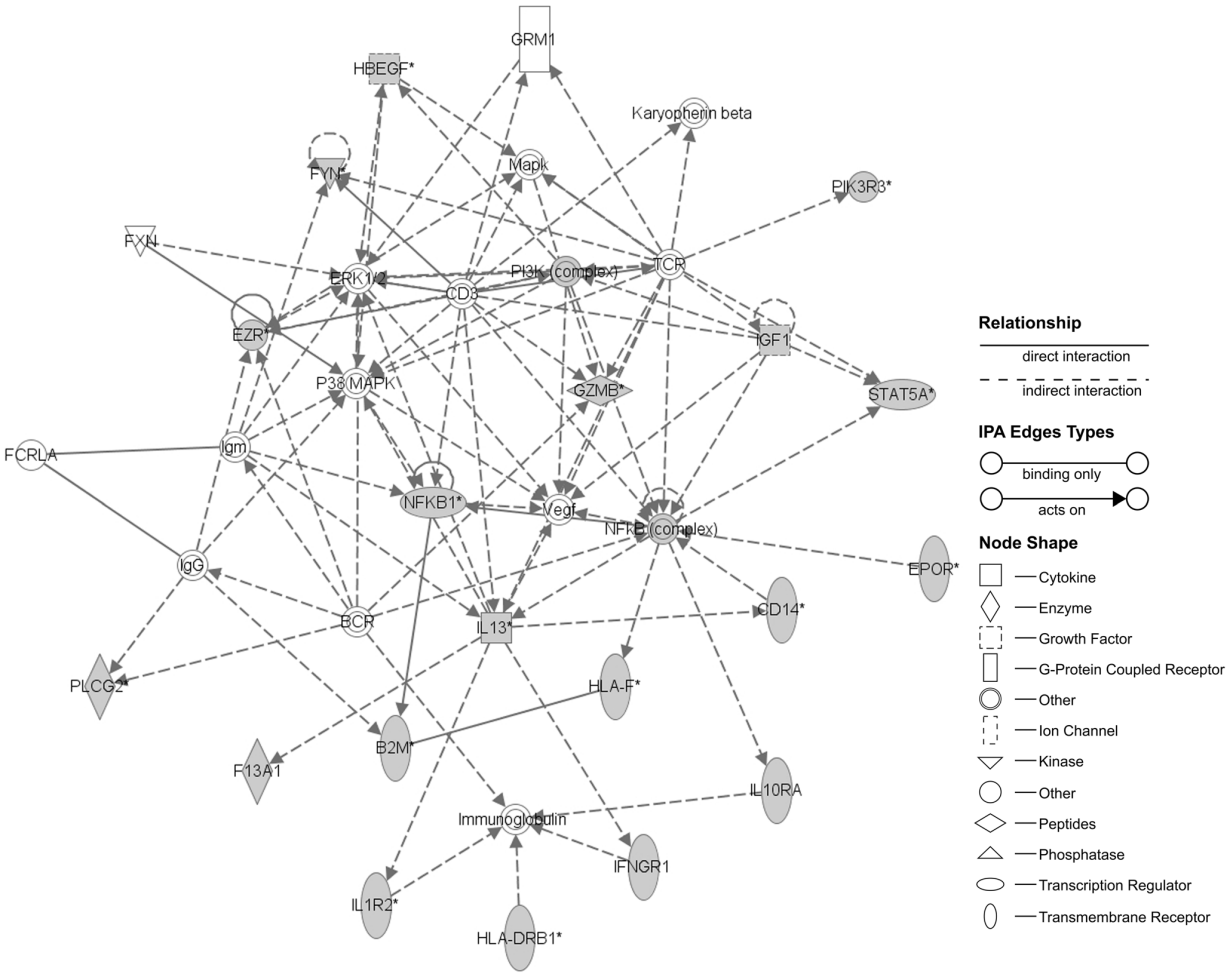
Ingenuity network analysis constructed using significant up-regulated genes (BH<0.05) founded during hASC culture evolution. The top network functions were Regulation of actin cytoskeleton, ECM-receptor interaction, cell division signaling pathway and ribosome. ABI1 = abl-interactor 1; ABL1 = c-abl oncogene 1, receptor tyrosine kinase; AKAP13 = A-kinase anchor protein 13; CASP8AP2 = caspase 8 associated protein 2; caspase = apoptosis -related cystein peptidase; CEBPA = CCAAT/enhancer-binding protein alpha; CHEK1 = CHK1 checkpoint homolog (S. pombe); CLEC11A = C-type lectin domain family 11; CRHR1 = corticotropin-releasing factor receptor 1; DYNLT3 = dynein light chain Tctex-type 3; ERK1/2 = mitogen activated protein kinase; FAS = Fas (TNF receptor superfamily, member 6); FGF2 = fibroblast growth factor 2 (basic); FN1 = fibronectin 1; GLO1 = glyoxalase I; IL36A = interleukin-36 alpha; IL36B = interleukin-36 beta; IL36G = interleukin-36 gamma; LITAF = lipopolysaccharide-induced TNF factor; MICA = MHC class I polypeptide-related sequence A; NFkB = NF-kappa-beta; P2RY6 = pyrimidinergic receptor P2Y, G-protein coupled, 6; PFN2 = profilin-2; Pkc(s) = protein kinase C; PPP2RA = protein phosphatase 2; RIN1 = ras and Rab interactor 1; RPLP0 = ribosomal protein, large, P0; RRAS2 = related RAS viral (r-ras) oncogene homolog 2; SATB1 = DNA-binding protein SATB1; SDC1 = syndecan 1; SMPD2 = sphingomyelin phosphodiesterase 2, neutral membrane (neutral sphingomyelinase); Sphk = sphingosine kinase; WASF1 = WAS protein family, member 1; WASF2 = WAS protein family, member 2; WASF3 = WAS protein family, member 3.The grey nodes are the genes classified as significant. The asterisk (*) indicates the degree of up-regulation.

**Figure 6:**
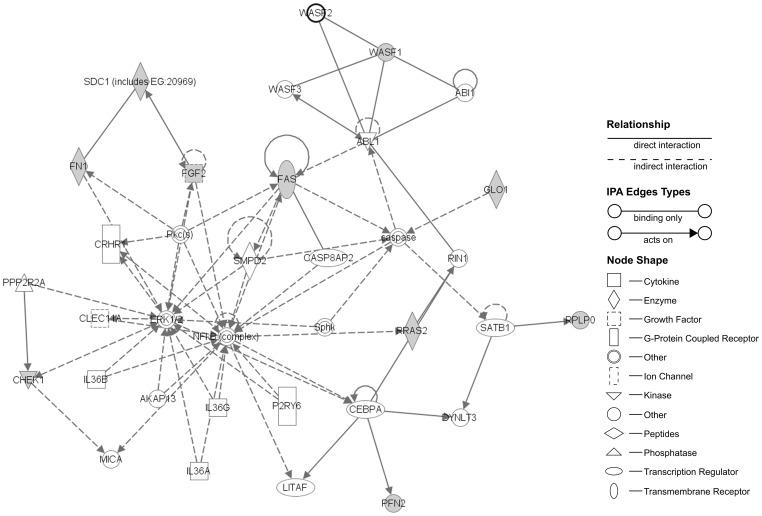
Ingenuity network analysis constructed using significant down-regulated genes (BH<0.05) founded during hASC culture evolution. The top network functions were Hematopoietic cell lineage, Cell adhesion molecules, Leucocyte transendothelial migration and Complement and coagulation cascades. B2M = beta-2-microglobulin; BCR = breakpoint cluster region; CD14 = monocyte differentiation antigen CD14; CD3 = T-cell surface glycoprotein CD3 epsilon chain; EPOR = erythropoietin receptor; ERK1/2 = mitogen activated protein kinase; EZR = ezrin; F13A1 = coagulation factor XIII, A1 polypeptide; FCRLA = Fc receptor-like A; FXN = frataxin, nuclear gene encoding mitochondrial protein; FYN = tyrosine-protein kinase Fyn; GRM1 = glutamate receptor, metabotropic 1; GZMB = granzyme B (granzyme 2, cytotoxic T-lymphocyte-associated serine esterase 1); HBEGF = heparin-binding EGF-like growth factor; HLA-DRB1 = MHC class II antigen HLA-DRB1 beta 1; HLA-F = HLA class I histocompatibility antigen, alpha chain F; IFNGR1 = Interferon gamma receptor 1; IGF1 = insulin-like growth factor 1 (somatomedin C); IL10RA = interleukin 10 receptor, alpha; IL13 = interleukin 13; IL1R2 = interleukin 1 receptor, type I; Immunoglobulin = Immunoglobulin; Karyopherin beta = nucleo cytoplasmic transporter; lgG = inmunoglobulin G; lgm = immunoglobulin M; Mapk = Mitogen-activated protein kinase; NFKB = NF-kappa-beta; NFKB1 = nuclear factor NF-kappa-B p105 subunit; P38MAPK = map kinase p38; PI3K = phosphatidylinositol 4-phosphate 3-kinase; PIK3R3 = phosphatidylinositol 3-kinase regulatory subunit gamma; PLCG2 = 1-phosphatidylinositol 4,5-bisphosphate phosphodiesterase gamma-2; STAT5A = signal transducer and activator of transcription 5B; TCR = T cell antigen receptor; VEGF = vascular endothelial growth factor. The grey nodes are the genes classified as significant. The asterisk (*) indicates the degree of down-regulation.

To further characterize possible changes or abnormalities induced in the cells by the increase of proliferation kinetics, we next studied their transcriptional profile evolution during culture with HS, using microarrays to compare possible variations of their profile in the different passages [Analysis and data available are provided at Text S1, and Table S2 and Figure S2]. Detailed results of the microarray gene expression in HS cultured cells, following comparison between passages, are shown in Tables 1, 2, 3, 4 and 5. Internal controls resulted as expected for each array. From a total of probes we paid attention to the intragroup gene expression differences between passages. Concerning significant up-regulation events (BH p-value <0.05), as shown on Table 1, we found 63 genes up-regulated on passage 1 versus the moment of isolation, 111 on passage 3 versus passage 1 and 209 on passage 5 versus passage 3. Regarding significant down-regulation (BH p-value <0.05), we detected 97 genes from passage 1 to passage 0, 136 from passage 3 to passage 1 and 237 from passage 5 to passage 3 (see Table 2, 3 and 4). Once the differences in the expression patterns between passages were obtained, we wanted to determine any possible relationships between the up- regulated and down-regulated genes, in order to identify any functional or molecular pathways involved. To determine such relations we used DAVID bioinformatics resources [42], [44]. KEGG was used to link the genes obtained with potential biological processes in which hASCs could be involved. The KEGG pathways found were compiled in Tables 1, 2, 3, 4 and 5. Summarizing, up-regulated genes (Table 1) observed when comparing cells processed immediately after isolation (G0) to their counterparts in passage 1, were mostly related with extracellular matrix (ECM) formation and biosynthesis of unsaturated fatty acids.Comparing p3 against p1, the biggest differences in expression were related with metabolism and cell cycle and the same between p5 and p3. Down-regulated genes (Table 2, 3 and 4) were involved in migration routes (passage 1 versus isolation) and immunomodulation (passage 3 versus passage 1, and passage 5 versus passage 3). Both the up- and down-regulated significant genes were highly related between them. In addition, the Ingenuity Core Analysis found two networks, one for up-regulated and another for down-regulated, with their associated functions. Up-regulated network (Fig. 5) showed that FN1, FGF2, RRAS2, PFN2, SDC1, RPLP0, CHEK1, FAS, WASF1 and GLO1 genes were significantly overexpressed. These genes were classified into several function categories (BH<0.05), the top were Regulation of actin cytoskeleton, ECM-receptor interaction, Cell division signaling pathway, Cell cycle and Ribosome. In the case of down-regulated network (Fig. 6) were HBEGF, FYN, PIK3R3, EZR, PI3K, IGF1, STAT5A, GZMB, NFKB1, NFkB, IL13, CD14, EPOR, HLA-F, B2M, PLCG2, F13A1, IL1R2, IFNGR1, HLA-DRB1 and IL10RA. These genes were grouped in functions as Hematopoietic cell lineage, Cell adhesion molecules, Leucocyte transendothelial migration and Complement and coagulation cascades. The networks included a subset of the predicted functional partners and their relationships whose could be considered to be involved in the maintenance and progression of hASC culture system.

**Table 5:** Genes showing expression changes in all passages that were involved in significant KEEG pathways.

Number of genes BH<0.05	KEGG Pathway	Genes in KEGG pathway	Fisher exact p-value
62	Neurotrophin signalling pathway	ARHGDIB, NTRK2,PIK3R3	4.2·10^−3^

## Discussion

Given the wide clinical potential that hASCs had demonstrated, our objective was to design a new protocol for the isolation and maintenance of clinical grade safe cells avoiding xenogeneic reagents without affecting their characteristics and multipotency. Use of animal origin reagents is a controversial issue to be taken into account, because of zoonosis disease transmission. The risk of prion infection can be avoided by using New Zealand FBS. However, use of an average of 20% FBS in cell culture, regardless of the type results in hMSCs carrying between 7 to 30 mg of bovine serum proteins [45], is able to elicit an immune reaction in patients [16], [46].

In addition, the expression of some murine derived syalic derivatives was observed in human cells that had been cultured in contact with mouse fibroblasts, with unknown implications [31].

On the other hand, differences in gene expression levels between MSCs cultured with FBS versus HS have been reported [47], [48]. This evidence supports the hypothesis that the use of FBS could affect cell behavior, differentiation or senescence, and increase the risk of instability. Several groups have tried to solve the issue by introducing modifications in the protocols such as the use of autologous HS [49], serum free media [50] or pooled human platelet lysate [4]. Previously, a protocol has been [46] developed to eliminate contamination with fetal calf serum, although this technique is overshadowed by the efficiency of human derivatives. Partial solutions to the problem were those in which FBS could be substituted during the isolation and maintenance of the cells by human AB serum from a donor pool [21] or the culture using synthetic substitutes [51]. Although these are great improvements, other animal agents were used during the isolation and culture, such as BSA or porcine-derived trypsin. In this context, our efforts are focused also on improving such protocol by introducing HSA and Tryple®, a non animal reagent that helps to detach cells. Tryple® has been widely employed in the literature for detaching and reseeding of cells. In our case, the cultures did not seem to be altered or compromised by its use and their karyotype remained normal up to passage 15 (data not shown).

The amendments introduced in our protocol during the isolation and culture did not compromise the viability, growth or characteristics of the hASCs obtained. Instead, they did improve some of these aspects. Use of HS induced high proliferative growth that increased steadily until passage 18 (33.9±4.1 HS versus 13.9±3.3 in FBS). Moreover, the introduction of HS did not interfere with the hASCs morphology, which retains a characteristic fibroblast–like morphology and cellular organization [12], [20]. This observation was then confirmed at an ultrastructural level through TEM.

The introduction of wash and spin cycles with HSA during the isolation, also proved to be beneficial, eliminating the majority of mature adipocytes.

In addition, we did not observe any increase in the number of mature adipocytes during the maintenance and expansion of the cell cultures due to uncontrolled differentiation, indicating that both the media and the conditions under which the crops are kept helped preserve cell multipotency. Regarding expression patterns, detectable levels of mesenchymal stem cell markers were still observed after expanding the cell cultures 5 times. This result confirmed that the conditions used for cell maintenance were adequate to preserve the undifferentiation of hASCs. The expression of hematopoietic stem cell markers such as CD45 or CD14, was also detected in the initial stages of isolation and culture in certain samples but decreased progressively throughout the passages. This observation was confirmed with the microarray analysis. At the same time, expression of adipocyte progenitor cell markers was detected in the culture during isolation and expansion. These observations indicate that the vast majority, if not all, of the stem cells present in the culture after the first passage, had a clear adipose origin and were not derived from hematopoietic stem cells.

Concerning differentiation studies, our results confirmed that hASCs cells obtained using our isolation protocol maintain their multipotent capacity. Detailed ultrastructure and gene analysis of differentiated cells using TEM revealed that their morphology was compatible with mature adipocytes and cartilage. In addition, we wanted to be sure that the culture conditions did not alter hASCs properties or induce malignization, so we performed microarray analysis. We observed that in case of up-regulated genes hASCs cell behavior followed the classical steps for in vitro culture adaptation. This observation correlates with the proliferation curve data, which indicates that during passage 5 hASCs are in exponential growth phase. In summary, expression patterns indicate that during the initial isolation phase hASCs first adhere to the culture surface, progressively adapt to the in vitro environment conditions and finally turn on the proliferation pathways. We did not detect up-regulation of pathways related with cell death or senescence in any of the passages. Down-regulation of gene expression was also analyzed. Immediately between isolation and the first passage a down regulation of genes related with migration and proliferation was observed. This result is coherent with the previous observation that adhesion pathways were instead up-regulated. Comparison between passage 3 versus passage 1, and passage 5 versus passage 3, allowed us to detect a reduction in the expression of biochemical pathways related with immune reaction. This observation can be explained both by the progressive elimination of blood cells through the sequential washing and passaging, and due to the fact that hASCs act in vitro and in vivo as immunomodulatory cells [22], [25], [26], [52], [53]. Microarray analysis allowed us to conclude that the changes introduced trough our protocol, did not compromise their viability, characteristics and potential.

### Conclusions

This is the first protocol for hMSCs performed without using any animal origin reagent. With this work we have proven that these amendments support the isolation and maintenance of hASCs, preserving or improving crucial culture parameters such as viability, cell morphology and identity during long term culture, as well as proliferation and differentiation ability. To our knowledge, this is the nearest good manufacture practice condition protocol developed for hASCs, and it is a low-cost method that can be routinely and easily used for clinic and research.

## Supporting Information

Figure S1 **Flow cytometry analysis and percentage of expression of mesenchymal stem cell markers in HS-hASCs versus FBS-hASCs.** The figures show the flow cytometry analysis of some mesenchymal (CD90, CD73, CD44) and hematopoietic (CD14, CD117, CD166, CD144) stem cell markers in hASCs (passage 5) cultured in A) HS and B) FBS respectively.
(PDF)

Figure S2 **Evolution of CD34 and CD45 gene expression in hASC cultures xenogeneic –free isolated and maintained.**
(PDF)

Table S1 **Primer sequences for the characterization of isolated hASCs.** PCR primers used in the study and conditions. All of them had been designed using Primer 3 tools. Cartilage differentiation primers have been performed by Zuk et al., 2002.
(PDF)

Table S2 **Microarray gene expression analysis.**
(XLS)

Text S1 **Supplementary Methods.**
(PDF)
