# Supplementary figures and images for: A Xenogeneic-Free Protocol for Isolation and Expansion of Human Adipose Stem Cells for Clinical Uses
Source: PLoS One. 2013 Jul 9;8(7):e67870. doi: 10.1371/journal.pone.0067870 (PMC3706484; doi:10.1371/journal.pone.0067870)

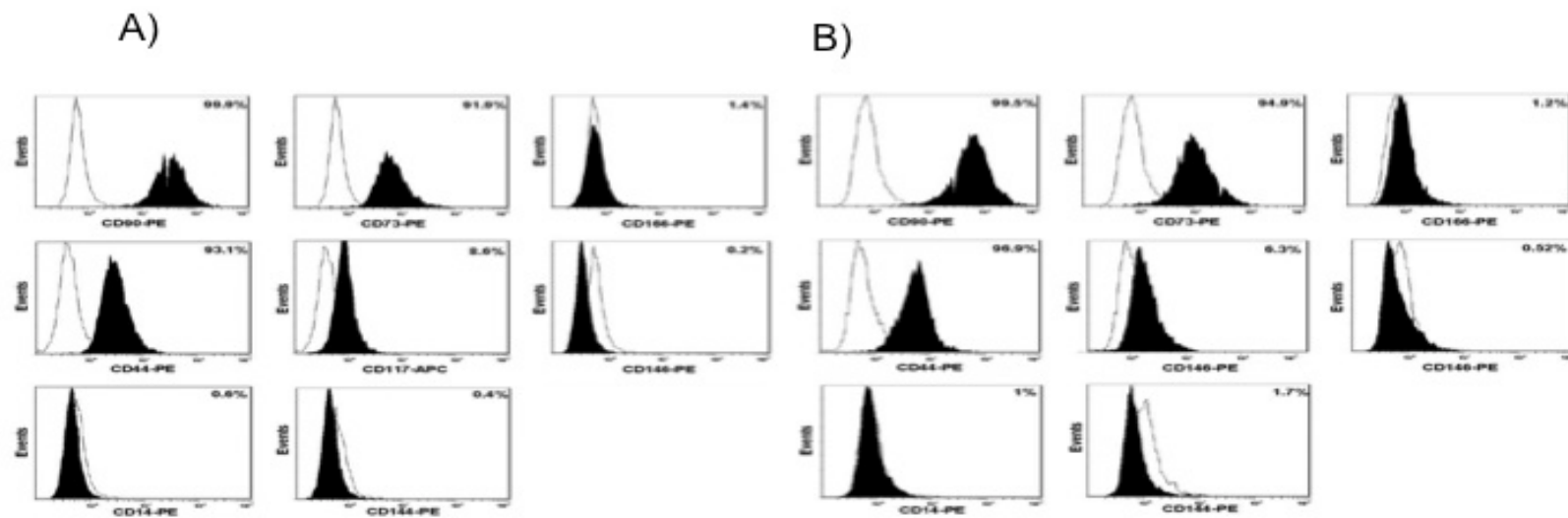

| MARKER | HUMAN SERUM | FETAL BOVINE SERUM |
|--------|-------------|--------------------|
| CD90   | 99.9%       | 99.5%              |
| CD73   | 91.9%       | 94.9%              |
| CD166  | 1.44%       | 1.2%               |
| CD44   | 93.1%       | 96.9%              |
| CD117  | 8.6%        | 6.3%               |
| CD146  | 0.2%        | 0.52%              |
| CD14   | 0.6%        | 1%                 |
| CD144  | 0.4%        | 1.7%               |

Supplement: Figure S1 — Flow cytometry analysis and percentage of expression of mesenchymal stem cell markers in HS-hASCs versus FBS-hASCs. The figures show the flow cytometry analysis of some mesenchymal (CD90, CD73, CD44) and hematopoietic (CD14, CD117, CD166, CD144) stem cell markers in hASCs (passage 5) cultured in A) HS and B) FBS respectively. (PDF) [file pone.0067870.s001.pdf]

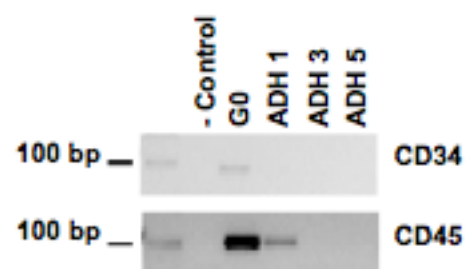

Supplement: Figure S2 — Evolution of CD34 and CD45 gene expression in hASC cultures xenogeneic –free isolated and maintained. (PDF) [file pone.0067870.s002.pdf]
