# Supplementary material for: A Xenogeneic-Free Protocol for Isolation and Expansion of Human Adipose Stem Cells for Clinical Uses
Source: PLoS One. 2013 Jul 9;8(7):e67870. doi: 10.1371/journal.pone.0067870 (PMC3706484; doi:10.1371/journal.pone.0067870)
Supplement: Table S1 — Primer sequences for the characterization of isolated hASCs. PCR primers used in the study and conditions. All of them had been designed using Primer 3 tools. Cartilage differentiation primers have been performed by Zuk et al., 2002. (PDF) [file pone.0067870.s003.pdf]

Gene	Primer sequencies	Annealing Temperature	Size	Detection	Source
**HOUSEKEEPING GENES**					
B-2Microglobulin	**F:**CTCGCGCTACTCTCTCTTTCTG<br>**R:**GCTTACATGTCTCGATCCCACT	55ºC	335 pb	RT-PCR<br>QRT-PCR	-
**MESENCHYMAL STEM CELLS GENES**					
CD90	**F**: **CGCTCTCCTGCTAACAGTCTT**<br>**R**: **CAGGCTGAACTCGTACTGGA**	60ºC	142 pb	QRT-PCR	-
CD73	**F:** CTTAACGTGGGAGTGGAACC<br>**R:** TCTAGCTGCCATTTGCACAC	60ºC	102 pb	QRT-PCR	-
CD105	**F**: CTAACTGGCAGGGGAGACAG<br>**R**: CTCCATGTGGCAGGAGCTA	60ºC	101 pb	QRT-PCR	-
CD166	**F:** ATTGAAGTTTTATTTGGCAGGAA<br>**R**: GGCTTAGCCATGCAAAACA	60ºC	102 pb	QRT-PCR	-
CD13	**F**: CTCTCGATGGACAATGAACG<br>**R:** ATCTGCTGCCCTGTTGATTC	60ºC	100pb	QRT-PCR	-
**HEMATOPOIETIC STEM CELLS GENES**					
CD14	**F:** TCCAAGACAGAATAATGAATGGAC<br>**R**: CGAAAAGTCCTCAACGTCCT	60ºC	76 pb	QRT-PCR	-
CD34	**F:** TCTGGATCAAAGTAGGCAGGA<br>**R**: GATCCAGCCTCAGAGGAAGA	60ºC	96 pb	RT_PCR	-
**DIFFERENTIATION ASSAY GENES**					
LPL	**F:**CAAAGCCCTGCTCGTGCTGA<br>**R:**CAGCCAGTCCACCACAATGA	60ºC	334 pb	RT_PCR	-
ADIPONECTIN	**F**:TCCTTACAGAACACGCTTTCA<br>**R**:AGGGCCACAGAACGAGAG	60ºC	501 pb	RT_PCR	-
FABP4/AP2	**F**: AATGGGATGGAAAATCAACCA<br>**R:**GTGGAAGTGACGCCTTTCAT	60ºC	88 pb	RT_PCR	-
PPARgamma	**F:**TCAGCGGGAAGGACTTTATGTATG<br>**R:**TCAGGTTTGGGCGGATGC	60ºC	147 pb	RT_PCR	-
PLIN	**F:**GCAGTCAACAAAGGCCTCAC<br>**R:**AAGCTACTGGCGCTCTGCAC	60ºC	200 pb	RT_PCR	-
SREBF1/ADD1	**F:**GGAGCCATGGATTGCACTTTC<br>**R:**ATCCTTCAATGGAGTGGGTGCAG	60ºC	261 pb	RT_PCR	-
LEPTIN	**F**: AATGCATTGGGGAACCCTGT<br>**R**: CCAGGTCGTTGGATATTTGG	60ºC	305 pb	RT_PCR	-
Aggrecan variant 1 (AG1). Cartilage differentiation	**F:** GCAGAGACGCATCTAGAAATT<br>**R:** GGTAATTGCAGGGAACATCAT	55ºC	502 pb	RT_PCR	Zuk *et al, 2002*
Aggrecan variant 2 (AG2). Cartilage differentiation	**F:**GCAGAGACGCATCTAGAAATT<br>**R:**GGTAATTGCAGGGAACATCAT	55ºC	616 pb	RT_PCR	Zuk *et al, 2002*
Decorin (DEC). Cartilage differentiation	**F:** CCTTTGGTGAAGTTGGAACG<br>**R**: AAGATGTAATTCCGTAAGGG	55ºC	306 pb	RT_PCR	Zuk *et al, 2002*
Biglycan. Cartilage differentiation	**F:** TGCAGAACAACGACATCTCC<br>**R:** AGCTTGGAGTAGCGAAGCAG	55ºC	346 pb	RT_PCR	Zuk *et al, 2002*
Collagen type X alpha1 (CN10). Cartilage differentiation.	**F:** TGGAGTGGGAAAAAGAGGTG<br>**R:** GTCCTCCAACTCCAGGATCA	55ºC	599 pb	RT_PCR	Zuk et al, 2002
