# Supplementary material for: A Xenogeneic-Free Protocol for Isolation and Expansion of Human Adipose Stem Cells for Clinical Uses
Source: PLoS One. 2013 Jul 9;8(7):e67870. doi: 10.1371/journal.pone.0067870 (PMC3706484; doi:10.1371/journal.pone.0067870)
Supplement: Text S1 — Supplementary Methods. (PDF) [file pone.0067870.s005.pdf]

SUPPLEMENTARY METHODS

Flow cytometry characterization.

Human adipose stem cells (n=3, passages 3–5) were characterized by flow cytometry (EPICS XL flow cytometer; Beckman Coulter, Fullerton, CA) using anti-human monoclonal antibodies directly conjugated to fluorescein isothiocyanate (CD15 [BD Pharmingen, CA, USA]), phycoerythrin (CD14, CD146,CD166, CD144 [R&D Systems, MN,USA], CD73 [BD] and CD90 [Beckman Coulter, Fullerton,CA]) or allophycocyanin (CD44, CD117[R&D]). Data acquisition and analysis were performed with Expo32 software (Beckman Coulter). The cells were labeled according to standard protocols. Matched labeled isotypes were used as controls.

Microarray data analysis.

Microarray Analysis. Hybridization, washing, staining and scanning of the arrays were performed according to the manufacturer's instructions (Agilent Technologies “One-Color Microarray-Based gene expression Analysis, 5.5 Version). The array contains 41000 genes.

Array Design.

Total RNA was obtained from lipoaspirates of eight donors (age 18-35 years) or isolated from $1.6x10^6$ cells in culture using the RNeasy kit (Qiagen), according to manufacturer’s instructions, and treated with DNAse. RNA concentration was measured by spectroscopy using Nanodrop and yielding an A_{260}/A_{280} ratio of 1:8. Before performing the Agilent hybridization procedure p/n 5188-5977 “one-color microarray-based gene expression analysis”, quality and quantity control of total RNA was assessed using electroforesis in Agilent Bioanalizer. The RNA Integrity Number (RIN) was between 8.0 and 9.9 in all the samples used for hybridization. Total **RNA was standardized among patients pooling together the different samples for each established stages.** Four time-points were established for the analysis (Isolation, passage 1, passage 3 and passage 5), using four primary culture RNA preparations from 4 different patients per time-point. The hybridization was repeated twice per group.

Microarray gene expression data analysis.

Statistical analysis was carried out using the R-project software (http://www.r-project.org/) and the appropriate Bioconductor packages (http://www.bioconductor.org/) run under R-project. First, background correction was carried out using the *subtract* function implemented in the *limma* package (48). Then, and in order to remove all the possible sources of variation of a non-biological origin between arrays, densitometry values between arrays were normalized using the *quantile* normalization function also implemented in the *limma* package.

Statistically significant differences between groups were identified using the empirical Bayes method, implemented in *limma* (48), to moderate the standard errors of the estimated log-fold changes, which results in more stable inference and improved power, especially for experiments with small numbers of arrays. This approach includes the Benjamini-Hochberg (BH) multiple hypothesis test for raw p-value correction to ascertain the false positive rate. Those genes showing a BH p-value<0.05 were selected as de-regulated genes.

Functional annotations were carried out using the Database for Annotation, Visualization and Integrated Discovery (DAVID) (23,43), which allows the searching for blocks of functionally-related genes by different criteria such as the Gene Ontology terms and KEGG pathways, among others.
